# DR5 mAb-conjugated, DTIC-loaded immuno-nanoparticles effectively and specifically kill malignant melanoma cells *in vivo*

**DOI:** 10.18632/oncotarget.11014

**Published:** 2016-08-02

**Authors:** Baoyue Ding, Wei Zhang, Xin Wu, Jeffrey Wang, Chen Xie, Xuan Huang, Shuyu Zhan, Yongxia Zheng, Yueyan Huang, Ningyin Xu, Xueying Ding, Shen Gao

**Affiliations:** ^1^ Department of Pharmaceutics, Medical College of Jiaxing University, Jiaxing, PR China; ^2^ Department of Pharmaceutics, Changhai Hospital, Second Military Medical University, Shanghai, PR China; ^3^ Department of Pharmaceutical Sciences, College of Pharmacy, Western University of Health Sciences, Pomona, USA; ^4^ Department of Pharmaceutics, Shanghai Pulmonary Hospital, Tongji University, Shanghai, PR China; ^5^ Department of Pharmaceutics, Shanghai General Hospital, Shanghai Jiaotong University, Shanghai, PR China

**Keywords:** malignant melanoma, DR5 monoclonal antibodies, dacarbazine, chemoimmunotherapy, active targeting nanoparticles

## Abstract

We combined chemo- and immunotherapies by constructing dual therapeutic function immuno-nanoparticles (NPs) consisting of death receptor 5 monoclonal antibody (DR5 mAb)-conjugated nanoparticles loaded with dacarbazine (DTIC) (DTIC-NPs-DR5 mAb). We determined the *in vivo* targeting specificity of DTIC-NPs-DR5 mAb by evaluating distribution in tumor-bearing nude mice using a real-time imaging system. Therapeutic efficacy was assessed in terms of its effect on tumor volume, survival time, histomorphology, microvessel density (MVD), and apoptotic index (AI). Systemic toxicity was evaluated by measuring white blood cells (WBC) counts, alanine aminotransferase (ALT) levels, and creatinine clearance (CR).*In vivo* and *ex vivo* imaging indicates that DR5 mAb modification enhanced the accumulation of NPs within the xenograft tumor. DTIC-NPs-DR5 mAb inhibited tumor growth more effectively than DTIC or DR5 mAb alone, indicating that combining DTIC and DR5 mAb through pharmaceutical engineering achieves a better therapeutic effect. Moreover, the toxicity of DTIC-NPs-DR5 mAb was much lower than that of DTIC, implying that DR5 mAb targeting reduces nonspecific uptake of DTIC into normal tissue and thus decreases toxic side effects. These results demonstrate that DTIC-NPs-DR5 mAb is a safe and effective nanoparticle formulation with the potential to improve the efficacy and specificity of melanoma treatment.

## INTRODUCTION

Malignant melanoma (MM) is the most aggressive form of skin cancer causing the majority of skin cancer-related deaths. Its incidence has increased in recent decades worldwide [1]. While adequate resection of the primary cutaneous melanoma leads to an excellent prognosis [2], the prognosis of metastatic melanoma is poor, with a five-year survival rate less than 6% and median survival time less than one year [3]. The most common treatment for the metastatic melanoma is chemotherapy; dacarbazine (DTIC) is the first-line drug [4]. However, DTIC therapy fails in 80-87% of patients [5], due to its rapid clearance following intravenous administration and the development of drug resistance. Consequently, the clinical application and therapeutic benefits of DTIC are limited [6–8]. Novel therapeutic strategies are urgently needed to improve patient outcomes for malignant melanoma [9, 10].

Its selective toxicity against malignancies makes TNF-related apoptosis inducing ligand (TRAIL) a promising anti-cancer candidate [11, 12]. TRAIL binds to death receptors 4 and 5 (DR4 and DR5), triggering an extrinsic apoptosis pathway by activating caspase-8 and subsequently, caspase-3 [13–15]. Many studies indicate that DR5 is highly expressed on the surface of MM cells and therefore can serve as a promising therapeutic target for MM [16–20]. In comparison to TRAIL, agonistic monoclonal antibody against DR5 (DR5 mAb) has been proven to possess potent *in vivo* antitumor activity with even better stability, lower clearance, and less toxicity [21–23]. In early phase clinical trials, monoclonal antibodies targeting DR5 (DR5 mAb) have also been shown to increase the cytotoxicity of conventional chemotherapeutic drugs [24–26].

Recent studies have focused on combining immune-based drugs, such as therapeutic monoclonal antibodies, with chemotherapeutic agents to achieve better MM treatments [27–29]. Given the difference in DR5 expression level between normal and MM cells, DR5 mAbs should be useful for targeted drug delivery. We developed a dual function immuno-nanoparticle formulation (DTIC-NPs-DR5 mAb) in our previous study with DTIC encapsulated on the inside and DR5 mAb covalently linked to the NP surface [30]. DTIC-NPs-DR5 mAb may possess not only the anti-cancer effects of DTIC and DR5 mAb, but also additional favorable pharmacokinetic properties, such as a long systemic circulation time and actively targeted distribution. In our previous *in vitro* studies, we demonstrated that DTIC-NPs-DR5 mAb specifically targeted and efficiently entered DR5 overexpressing MM cells. Additionally, these immuno-nanoparticles enhanced tumor cytotoxicity, increased cell apoptosis, and decreased non-specific toxicity.

In the current study, the *in vivo* targeting and therapeutic effect as well as the non-specific toxicity of DTIC-NPs-DR5 mAb were assessed using a malignant melanoma xenograft mouse model. Phosphate buffered saline (PBS), DTIC, DR5 mAb, blank nanoparticles (Blank-NPs), DTIC-loaded nanoparticles (DTIC-NPs) and DR5 mAb-conjugated blank nanoparticles (Blank-NPs- DR5 mAb) were used as controls.

## RESULTS

### Characterization of the nanoparticles

As shown in Table 1, the mean diameter of DTIC-NPs-DR5 mAb was 170.0 ± 4.1 nm, with an acceptable polydispersity index (PDI) of less than 0.3, and a zeta potential of −34.6 ± 2.3 mV. The morphology of the DTIC-NPs-DR5 mAb was determined as described in our previous study [30], they were found to be spherically shaped and moderately uniform. Drug loading (DL) and encapsulation efficiency (EE) of DTIC were 17.8 ± 0.8 μg/mg and 71.7 ± 2.5%, respectively. The amount of DR5 mAb conjugated to the nanoparticles was approximately 12.8 ± 2.4 μg DR5 mAb/mg nanoparticles as quantified by a protein assay.

**Table 1:** Characterization and drug content of DTIC-NPs-DR5 mAb (n=3)

Size (nm)	PDI	Zeta potential (mV)	DL of DTIC (μg/mg)	EE of DTIC (%)	DL of DR5 mAb (μg/mg)
170.0 ± 4.1	0.29	−34.6 ± 2.3	17.8 ± 0.8	71.7 ± 2.5	12.8 ± 2.4

### Dynamic and specific distribution of the NPs *in vivo*

**Figure 1:**
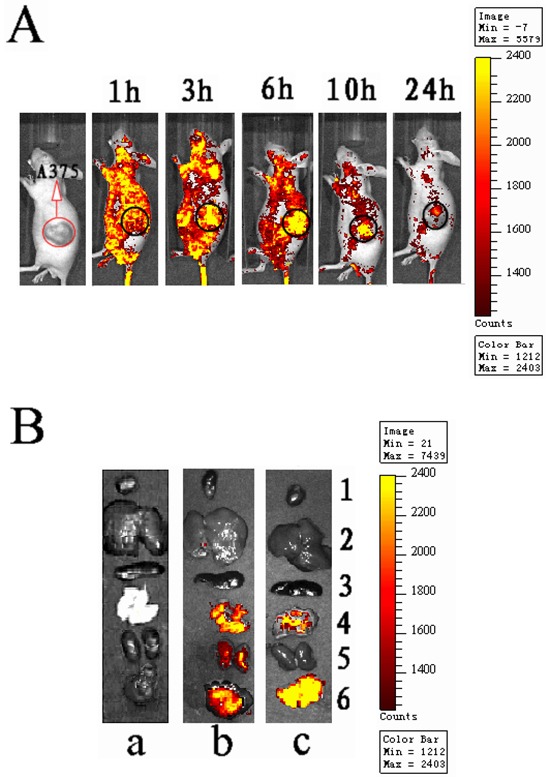
*In vivo* dynamic and specific distribution of antibody modified NPs **A.** *Real-time in vivo* bioluminescence imaging of PE-NPs-DR5 mAb-FITC in tumor-bearing nude mice at different time points (intravenously inject with 5 mg PE-NPs-DR5 mAb-FITC). **B.** Representative fluorescence images of dissected organs of nude mice bearing MM sacrificed 10 h after intravenous injection of different nanoparticles. **a:** Blank-NPs (control group); **b:** PE-NPs; **c:** PE-NPs-DR5 mAb-FITC. 1.heart; 2.liver; 3.spleen; 4.lung; 5.kidneys; 6.tumor. All images were acquired under the same conditions (5 mg/ml, 0.2ml NPs per mouse).

The mAb-FITC modified nanoparticles encapsulating a fluorescence agent (PE) were intravenously injected into tumor-bearing mice via the tail vein. Visible fluorescent distribution of PE in tumor-bearing mice was observed for 24 h using the IVIS Lumina II small animal *in vivo* real-time imaging system (Figure 1A). At 3 h post administration, a clear PE signal was visible at the tumor site. The signal reached its maximum at 6 h, and then gradually decreased. This suggests that the PE-NPs-DR5 mAb-FITC distribute into tissues in a time-dependent manner.

Tumors and other organs were excised and observed by *ex vivo* imaging 10 h after intravenous injection. As shown in Figure 1B, fluorescence signals of the unmodified NPs were observed in the tumor, lung and kidneys. Compared to unmodified NPs, the fluorescence signal of mAb-NPs was stronger in the tumor, weaker in the lung and not observed in the kidneys. This finding indicates that the mAb-NPs more specifically accumulated in tumor tissue *in vivo* than simple NPs did.

### *In vivo* antitumor efficacy

**Figure 2:**
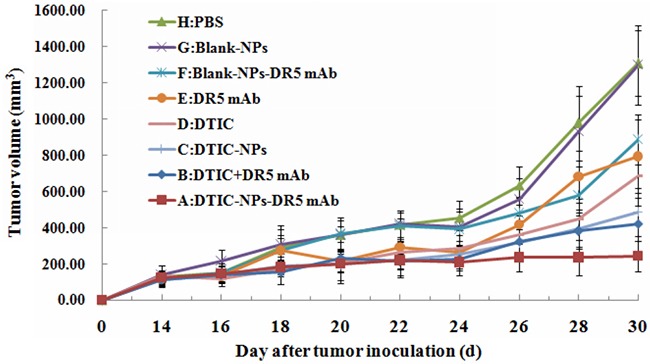
Growth curve of tumor in nude mice in therapeutic experiments Antitumor agents were given on the 14th day. A: DTIC-NPs-DR5 mAb group. B: DTIC + DR5 mAb group. C: DTIC-NPs group. D: DTIC group. E: DR5 mAb group. F: Blank-NPs-DR5 mAb group. G: Blank-NPs group. H: PBS group.

**Figure 3:**
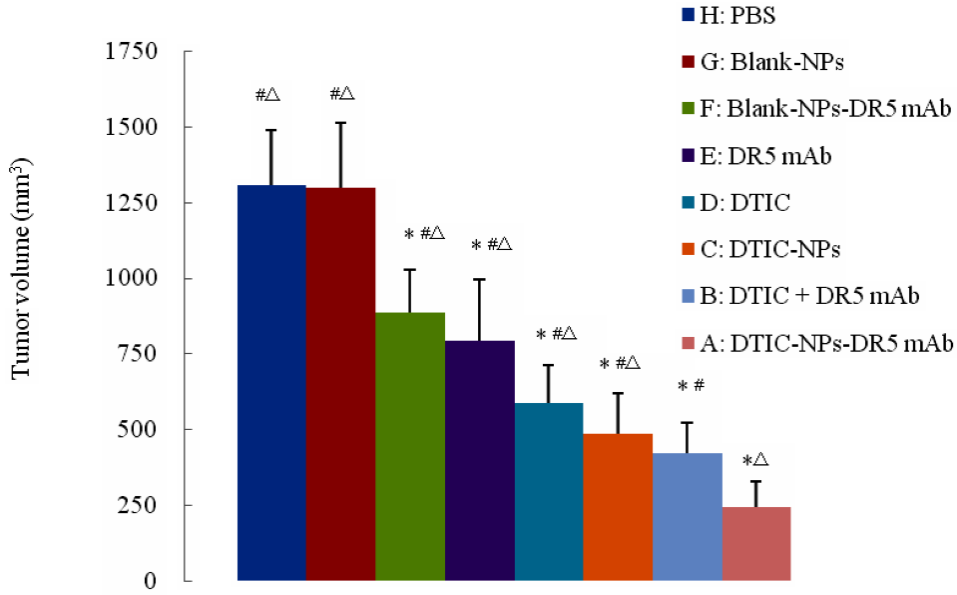
Final tumor volume of tumor-bearing nude mice in therapeutic experiments ^#^*p*<0.05, compared with DTIC-NPs-DR5 mAb (group A). ^Δ^*p*<0.05, compared with DTIC+DR5 mAb (group B). *p<0.05, compared with blank control group (group H).

**Figure 4:**
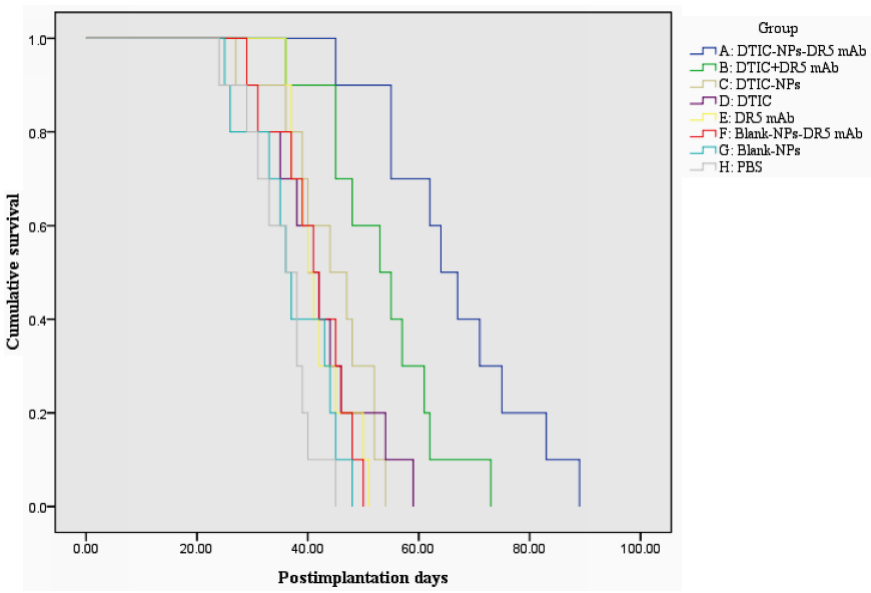
Kaplan-Meier survival curve of tumor-bearing nude mice in therapeutic experiments A: DTIC-NPs-DR5 mAb group. B: DTIC + DR5 mAb group. C: DTIC-NPs group. D: DTIC group. E: DR5 mAb group. F: Blank-NPs-DR5 mAb group. G: Blank-NPs group. H: PBS group.

Tumor weight and volume were determined for the evaluation of antitumor effect. Figure 2 shows the change of tumor volume with time (day) when treated with different agents. It is obvious that the animals treated with DTIC-NPs-DR5 mAb (Group A) show the slowest tumor growth rates and smallest tumor volumes. As shown in Table 2 and Figure 3, compared to PBS control (Group H), no significant change of tumor volume was observed in animals receiving Blank-NPs (Group G), and the tumor volume in the drug administered groups (Groups A-F) was significantly decreased (*p<0.05*). Moreover, the inhibition of tumor volume using the combination of DITC and mAb (Groups A and B) was superior to monotherapy with DITC or mAb (*p<0.05*) (Groups C-F). Notably, DTIC-NPs-DR5 mAb treatment exhibited the most significant suppression of tumor growth. Compared with the control Group H, all drug administered groups show significant prolongation of survival time except the DTIC and Blank-NPs groups (*p<0.05*) (Table 3 and Figure 4). The longest mean median survival was observed in the DTIC-NPs-DR5 mAb group.

**Table 2:** Average tumor weight and tumor growth inhibition rate (n=6)

Group	Average tumor weight (g)	Tumor growth inhibition rate (%)
A	0.29±0.19^Δ^^*^	81.32
B	0.58±0.20^#^^*^	63.45
C	0.82±0.25^#^^Δ^^*^	48.10
D	0.89±0.11^#^^Δ^^*^	43.53
E	0.94±0.13^#^^Δ^^*^	40.13
F	1.01±0.18^#^^Δ^^*^	35.68
G	1.44±0.27^#^^Δ^	8.65
H	1.57±0.35^#^^Δ^	—

# *p*<0.05, compared with DTIC-NPs-DR5 mAb (group A).

Δ *p*<0.05, compared with DTIC+DR5 mAb (group B).

* *p*<0.05, compared with blank control group (group H).

**Table 3:** Means and medians for survival time and the pairwise comparisons analyzed by the Kaplan-Meier method (n=10)

Group	Mean (days)	Median (days)	Pairwise comparisons for survival time		
			Compared with group A	Compared with group B	Compared with group H
A	66.6±4.2	64.0	-	^Δ^	^**^
B	53.5±3.3	53.0	^#^	-	^**^
C	43.9±2.7	44.0	^##^	^Δ^	^**^
D	41.8±1.7	40.0	^##^	^ΔΔ^	*p*<0.05
E	41.6±3.1	40.0	^##^	^Δ^	^*^
F	40.8±2.2	41.0	^##^	^ΔΔ^	^*^
G	37.2±2.5	36.0	^##^	^ΔΔ^	*p*<0.05
H	35.3±1.9	36.0	^##^	^ΔΔ^	-

# *p*<0.05,

## *p*<0.01, compared with DTIC-NPs-DR5 mAb (group A).

Δ *p*<0.05,

ΔΔ *p*<0.01, compared with DTIC+DR5 mAb (group B).

* *p*<0.05,

** *p*<0.01, compared with blank control group (group H).

### Histological examination, apoptosis index, and vascular count

**Figure 5:**
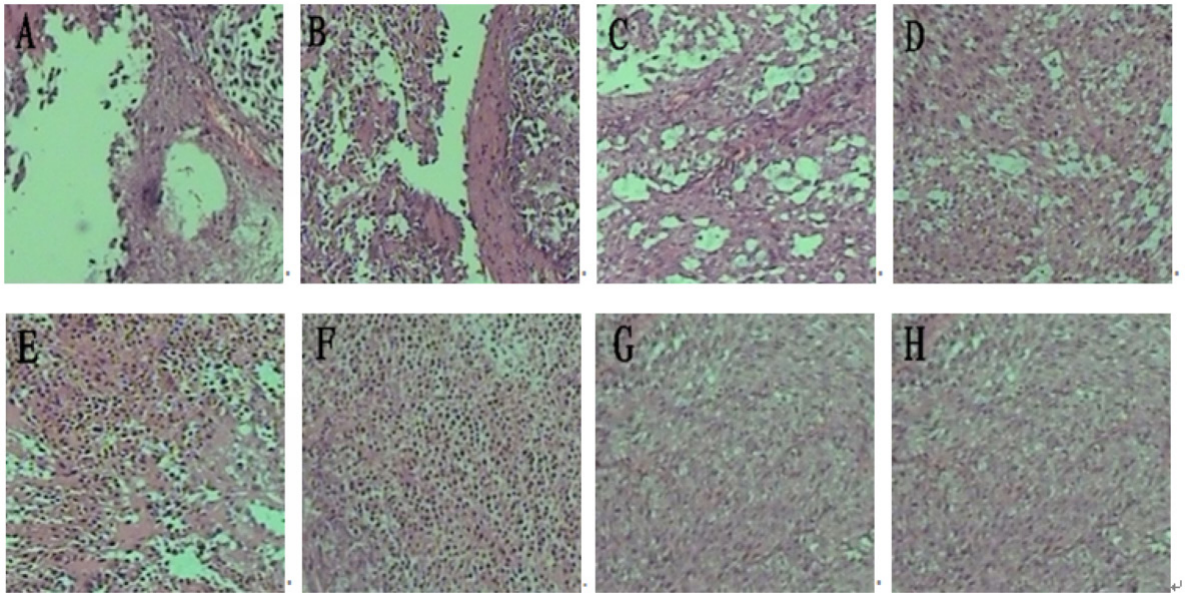
H&E histological staining of tumor sections from mice injected with different formulations **A.** DTIC-NPs-DR5 mAb group. **B.** DTIC + DR5 mAb group. **C.** DTIC-NPs group. **D.** DTIC group. **E.** DR5 mAb group. **F.** Blank-NPs-DR5 mAb group. **G.** Blank-NPs group. **H.** PBS group.

For a morphological characterization of the antitumor effect, hematoxylin-eosin staining was used to stain the tumors sections from all groups (Figure 5). High tissue density and vigorous cell growth in tumor tissue were observed in the PBS and Blank-NPs groups. The tissue density decreased in the DTIC treatment (groups C and D) and DR5 mAb treatment (groups E and F). In the combined therapy (groups A and B), cell proliferation decreased and slightly fibrous tissues were observed. In particular, large necrotic regions and extensive necrotic fibrous tissue inside the tumor was detected in Group A with DTIC-NPs-DR5 mAb treatment, indicating the dual therapeutic functional nanoparticles (DTIC-NPs-DR5 mAb) possessed tumoricidal activity.

**Figure 6:**
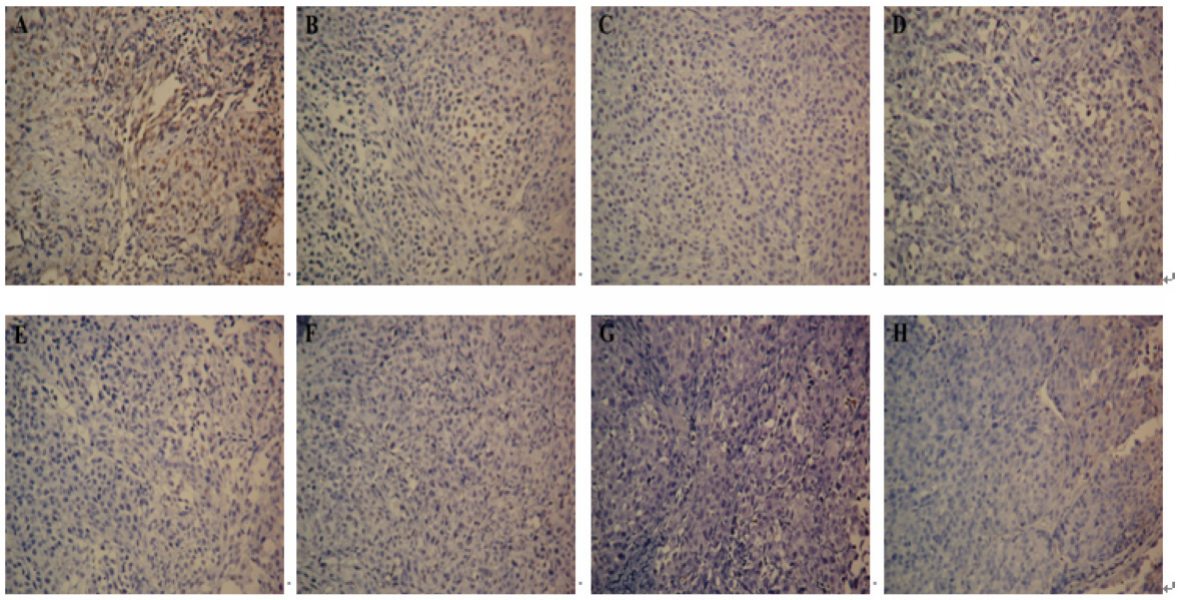
Apoptosis measurement of tumor sections from mice injected with different formulations: TUNEL assay(×200) **A.** DTIC-NPs-DR5 mAb group. **B.** DTIC + DR5 mAb group. **C.** DTIC-NPs group. **D.** DTIC group. **E.** DR5 mAb group. **F.** Blank-NPs-DR5 mAb group. **G.** Blank-NPs group. **H.** PBS group.

**Figure 7:**
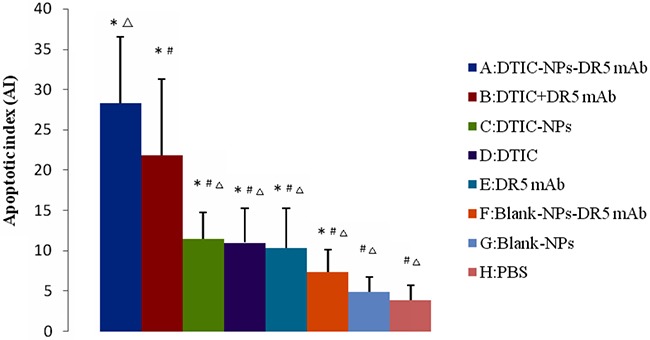
Apoptosis measurement of tumor sections from mice injected with different formulations: quantification of the apoptotic cells (n=6) The apoptotic index (AI) represents the number of TUNEL^+^ cells per 100 cells (n =6). ^#^*p*<0.05, compared with DTIC-NPs-DR5 mAb (group A). ^Δ^*p*<0.05, compared with DTIC+DR5 mAb (group B). *p<0.05, compared with blank control group (group H).

TUNEL staining was carried out to analyze intra-tumoral apoptosis. In the treatment groups (Groups A - F), apoptotic cells and apoptotic bodies within the cells were observed in the immunohistochemical staining images (Figure 6). DTIC-NPs-DR5 mAb and DTIC+DR5 mAb were more effective in inducing apoptosis than other treatment formulations (B-F) (*p*<0.05), and DTIC-NPs-DR5 mAb was superior to DTIC+DR5 mAb (Figure 7).

**Figure 8:**
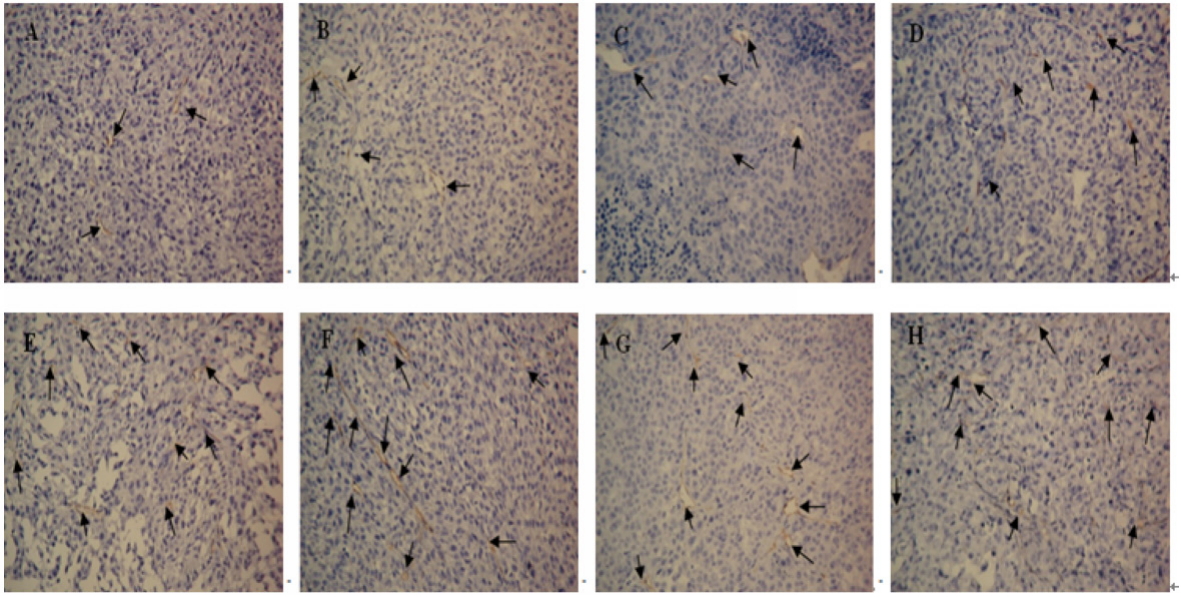
CD34 immunohistochemistry of tumor sections from mice after injection with different formulations: CD34 immunohistochemistry(×200) **A.** DTIC-NPs-DR5 mAb group. **B.** DTIC + DR5 mAb group. **C.** DTIC-NPs group. **D.** DTIC group. **E.** DR5 mAb group. **F.** blank-NPs-DR5 mAb group. **G.** blank-NPs group. **H.** PBS group. The arrow indicates the microvessel.

**Figure 9:**
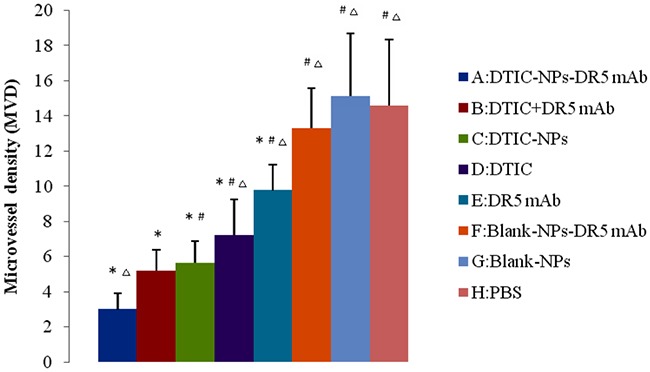
CD34 immunohistochemistry of tumor sections of mice after injection with different formulations: microvessel density (MVD) in the tumor sections (n=6) ^#^*p*<0.05, compared with DTIC-NPs-DR5 mAb (group A). ^Δ^*p*<0.05, compared with DTIC+DR5 mAb (group B). *p<0.05, compared with blank control group (group H).

Suppression of tumor vasculature was assessed through the detection of MVD as shown in Figures 8 and 9, the suppressive effect of DTIC-NPs-DR5 mAb (Group A) on tumor angiogenesis was more significant than other groups except the DTIC+ mAb (Group B) (*p*<0.05).

### Systemic toxicity

The most common side-effects of DTIC are marrow suppression, and hepatic and renal dysfunction. To evaluate the *in vivo* toxicity of the formulations, white blood cell number (WBC), alanine aminotransferase (ALT) level and creatinine clearance (CR) in the blood of the tumor-bearing nude mice were determined after treatment. As shown in Table 4, the WBC in the groups receiving DTIC solution (Groups B and D) was significantly lower than that in the PBS control group (Group H) and normal control group (*p*<0.05). Meanwhile, DTIC embedded into NPs (Groups A and C) resulted in a normal WBC value, indicating that the pharmaceutical engineering could reduce the marrow suppression by DTIC. Similar results were obtained on ALT. Compared with the control group (Group H) and normal control group, the ALT level was lower in the DTIC solution groups (Groups B and D) (*p*<0.05), and normal in the DTIC-NPs-DR5 mAb group (Group A). There was no significant change in CR in any groups. These findings indicate that the DTIC-NPs-DR5 mAb can alleviate the side-effects of DTIC.

**Table 4:** Test results of the white blood cells number (WBC), alanine aminotransferase (ALT) data and creatinine clearance (CR) of tumor-bearing nude mice after the treatment (n = 6)

Group	WBC(×10^9^/L)	ALT(u/L)	CR(μmol/L)
A	2.77±0.29	38.25±4.03	9.33±2.34
B	2.31±0.46^*^^#^	43.50±11.40^*^^#^	7.00±2.19
C	2.57±0.36	38.00±8.94	8.18±3.66
D	2.50±0.33^*^^#^	41.17±11.30^*^^#^	9.33±1.51
E	2.92±0.54	37.50±3.70	8.17±2.48
F	3.18±0.84	35.33±12.9	7.25±0.50
G	3.40±0.98	35.33±7.12	10.00±1.83
H	3.51±1.54	36.50±6.45	9.50±1.82
Normal control	3.66±0.51	35.66±5.27	9.17±2.86

* *p*<0.05, compared with blank control group (group H).

# *p*<0.05, compared with normal control group (healthy nude mice)

## DISCUSSION

In this study, we prepared therapeutic function immuno-nanoparticles (DTIC-NPs-DR5 mAb) and evaluated them *in vivo*. DTIC plays a role for tumoricidal chemotherapeutic activity, and their release from the nanoparticles can be controlled [30]. DR5 mAb on the NP surface can trigger apoptotic cell death providing additional tumoricidal effects on top of DTIC. Furthermore, DR5 mAb also acts as an active targeting device for the nanoparticles recognized by melanoma cells.

The distribution and accumulation results show that the unmodified NPs non-specifically distributed in various tissues, while the DR5 mAb-NPs more specifically accumulated in tumor tissue. The unmodified NPs possess a passive targeting property due to the EPR effect, which may lead to their accumulation in tumor tissue to a certain extent. The incorporation of DR5 mAb further improved the tumor targeting effect by recognizing and binding to the over-expressed TRAIL-R2 in melanoma cells. Therefore, the mAb-NPs can actively target the melanoma cells resulting in an increased level of intracellular delivery of the NPs.

The *in vivo* antitumor results indicated that the combination of DTIC and DR 5 mAb has an enhanced tumoricidal effect, as the DTIC+ mAb group, significantly inhibited tumor growth relative to the DTIC or DR5 mAb monotherapy or DTIC loaded in NPs. In a previous study, the combination of the DTIC +lexatumumab (agonistic TRAIL receptor −2 antibody) increased the sensitivity of tumor cells to the cytotoxic effect of DTIC or antibody, and reduced the growth of FEMX-1 xenografts *in vivo* compared with single drug [31]. The tumor volume of the mice treated with the DTIC-NPs-DR5 mAb formulation was significantly reduced compared to the DITC+ DR5 mAb (*p*<0.05), indicating that this pharmaceutical engineering not only obtained the combined antitumor effect of DITC+ DR5 mAb, but that active targeting of the delivery carrier provided an additional advantage. In addition, DTIC-NPs-DR5 mAb also showed a controlled release property, which further prolongs survival time.

H&E staining reveals that DTIC-NPs-DR5 mAb treatment significantly induced tumor cell necrosis and apoptosis, leading to superior suppression of tumor progression relative to other groups. TUNNEL assay results shows that the effect of Blank-NPs-DR5 mAb on tumor apoptosis was similar to that of DR5 mAb, indicating that the conjugation of DR5 mAb to the NPs has no effect on the activity of DR5 mAb. In contrast to monotherapy with DTIC or DR5 mAb, the combination of DTIC and DR5 mAb (Groups A and B) shows an additive and synergistic effect on apoptosis, which may due to the induced apoptosis by the intrinsic pathway of DTIC and extrinsic pathway of DR5 mAb. The CD34 immunohistochemistry study was conducted to evaluate neovascularization. MVD in the DTIC-NPs-DR5 mAb group was relatively lower compared with other groups. This may be due to the cell necrosis and apoptosis caused by the combination of DTIC and DR5 mAb, thereby reducing tumor growth. The systemic toxicity results showed that DTIC caused the marrow and hepatic toxicity. When DTIC was encapsulated within the DTIC-NPs-DR5 mAb formulation, its toxicity was reduced, possibly due to the reduced distribution of NPs in normal tissues by the active targeting effect and the decreased plasma concentration of DTIC through controlled drug release. DTIC showed antitumor effect, but had little effect on survival time, possibly due to its serious side-effects.

To summarize, we developed a novel dual function immuno-nanoparticle preparation, DTIC-NPs-DR5 mAb, which contains DTIC on the inside and covalently conjugated with the DR5 mAb on the outside. *In vivo* and *ex vivo* studies indicates that DTIC-NPs-DR5 mAb have an improved therapeutic efficacy with lower toxicity. Therefore, these immuno-nanoparticles offer a promising formulation for the treatment of melanoma.

## MATERIALS AND METHODS

### Materials

DTIC was purchased from Suzhou Lixin Pharmaceutical Co. (Suzhou, China). Methoxy poly (ethylene glycol)-poly (lactide) (MPEG-PLA) (3000:30000, Mw=33,000) was obtained from Shandong Institute of Medical Instruments (Shangdong, China). Humanized anti-DR5 mAb and FITC-labeled anti-DR5 mAb were purchased from Santa Cruz Biotechnology, Inc. (CA, USA). The human malignant melanoma cell line A375 was maintained in the International Joint Cancer Institute, Second Military Medical University (Shanghai, China). Dulbecco's Modified Eagle's Medium and fetal bovine serum were purchased from Hyclone (Logan, UT, USA). CD34 mouse anti-human monoclonal antibody was obtained from Maixin Biotechnology Development Co., Ltd. (Fuzhou, China). TUNEL apoptosis assay kit and IHC detection kit were purchased from Boehringer Mannheim GmbH. (Mannheim, Germany). Male BALB/c mice (4-6 weeks old) weighing 19-20 g were obtained from Shanghai Experimental Animal Center of the Chinese Academy of Sciences (Shanghai, China), and housed in groups under standard housing conditions. All experimental procedures were approved by the Animal Care and Use Committee of the Second Military Medical University. All other chemicals used in this work were of analytical grade and used as received.

### Preparation of nanoparticles

DTIC-NPs-DR5 mAb were prepared by a two-step procedure as described previously [30]. Briefly, DTIC-NPs were prepared using the double emulsion (w/o/w) and solvent evaporation method. Subsequently, DR5 mAb was conjugated to DTIC-NPs with EDC as the coupling agent. After preparation, the product was lyophilized. Control preparations, including DTIC-NPs, Blank-NPs-DR5 mAb, PE-NPs (phycoerythrin-loaded NPs), and PE-NPs-DR5 mAb-FITC (DTIC-NPs conjugated with FITC-labeled DR5 mAb) were obtained in a similar manner.

### Establishment of A375 BALB/c nude mouse tumor model

Tumor implantation was carried out by injecting 1×10^6^ A375 cells (5.0 × 10^6^ cells/ml, 0.2 ml per mouse) into the back subcutaneous region of each BALB/c nude mouse. The procedure was performed in a laminar flow hood using aseptic techniques. Tumor growth after implantation was monitored daily until Day 14. Mice with tumor size of approximately 150 mm^3^ were selected for the study.

### Evaluation of *in vivo* tumor targeting effect

To evaluate the *in vivo* real-time distribution and tumor targeting effect of DTIC-NPs-DR5 mAb in tumor-bearing nude mouse, 5 A375 malignant melanoma bearing nude mice were randomly selected and anesthetized. One mouse was injected intravenously with PE-NPs-DR5 mAb-FITC (5mg/ml, 0.2ml) via the tail vein, and another untreated mouse was used as the negative control. Both mice were placed in the observation room of the IVIS Lumina II small animal *in vivo* imaging system after l, 3, 6, 10 and 24h for real-time observation. The other three mice were given intravenous Blank-NPs, PE-NPs and PE-NPs-DR5 mAbs-FITC (5mg/ml, 0.2ml) via the tail vein. After 10 h, all mice were sacrificed and major organs and the tumor were removed immediately for imaging using the same imaging system.

### Animal groups and dosage regimens

To examine the therapeutic effect of DTIC-NPs-DR5 mAb *in vivo*, 48 A375 malignant melanoma-bearing nude mice were randomly divided into eight groups of 6 mice as follows: Group A (DTIC-NPs-DR5 mAb treatment group); Group B (DTIC + DR5 mAb treatment group); Group C(DTIC-NPs treatment group); Group D (DTIC treatment group); Group E (DR5 mAb treatment group); Group F (Blank-NPs-DR5 mAb treatment group); Group G (Blank-NPs treatment group); Group H (Blank control group). DTIC-NPs-DR5 mAb were administered once at a dosage of 2.5mg/kg by tail vein injection on Days 1, 3, 5 and 7. The dosage of the other treatment groups was adjusted to be equal to DTIC or DR5 mAb in the DTIC-NPs-DR5 mAb group. Group H received PBS as a blank control.

### Determination of *in vivo* antitumor effect

After dose administration, the therapeutic effect was evaluated by tumor growth suppressive and survival time. The tumor size was measured with a caliper every other day. Tumor volumes were calculated by the formula: V = length × width^2^/2. The mice were sacrificed by cervical dislocation and blood was collected after 16 day-treatment. Tumors were stripped and weighed to calculate the inhibition rate using the following formula: tumor weight inhibition rate (%) = (average tumor weight of the PBS control group - average tumor weight of drug treatment group / average tumor weight of the PBS control group) × 100%.

Another 80 tumor-bearing mice were randomly divided into 8 groups and then treated as above. The mice were observed daily until death. The survival time was recorded and analyzed.

### Histochemical and immunohistochemical analysis of tumor tissue

The tumor tissues, obtained from the *in vivo* experiment, were fixed, dehydrated, embedded, and cut into serial sections, one of the sections was stained with haematoxylin and eosin (H&E) using a standard protocol for tissue histological examination under light microscope.

Tumor apoptosis was measured by the TUNEL assay. Briefly, paraffin sections of the tumor tissue from each group were dewaxed with xylene and washed with PBS (pH 7.4) three times. The sections were digested with proteinase K for 15 min, blocked by 3% H_2_O_2_ for 30 min, added with the TUNEL mixed reaction liquid and then incubated a wet box at 40 °C overnight. After a 15 min incubation with POD anti-fluorescein antibody at 37°C, the sections were visualized using Converter-POD with diaminobenzidine (DAB) and subsequently counter-stained with hematoxylin. The sections were observed under a light microscope and the TUNEL^+^ cells, which distributed homogeneously within sight were counted. The apoptotic index (AI) represents the number of TUNEL^+^ cells per 100 cells.

To investigate the intratumoral microvessel density (MVD), three tumor tissue sections were used for immunohistochemical staining (S-P method) with a monoclonal anti-mouse CD34 antibody. The staining was carried out using routine operating procedures. The microvessels were confirmed as CD34 positive staining area and counted under microscope. In case of the unknown pathological features, the highest measured vascular density area was selected at low magnification. Microvessels were counted from five fields of view, and then the average value of microvessel counts was calculated.

### Toxicity study

To assess the adverse effect of dif determined using two-sample *t* test and the analysis of variance (ANOVA) using the SPSS19.0 software with P<0.05 as a significance level. Survival analyze was conducted using the Kaplan-Meier method and the comparison of groups was analyzed with the Log-rank.
